# Solution Blow Spun Mats with Beaded-Fiber Morphologies as a Drug Delivery System with Potential Use for Skin Wound Dressing

**DOI:** 10.1021/acsami.4c16675

**Published:** 2025-04-10

**Authors:** Javier Mauricio Anaya-Mancipe, Aline Luiza Machado Carlos, João Victor Dias de Assumpção Bastos, Elena Maria Tovar Ambel, Guillermo Velasco-Díez, Rosana Lopes Fialho, Rossana Mara da Silva Moreira Thiré

**Affiliations:** † Program of Metallurgical and Materials Engineering−PEMM/COPPE, Universidade Federal do Rio de Janeiro (UFRJ), 21941-598 Rio de Janeiro, RJ, Brazil; ‡ Department of Biochemistry and Molecular Biology, School of Biology, Universidad Complutense de Madrid−UCM, 28040 Madrid, Spain; § Instituto de Investigaciones Sanitarias San Carlos (IdISSC), 28040 Madrid, Spain; ∥ Post-graduation Program in Industrial Engineering, Polytechnic School, Universidade Federal da Bahia (UFBA), 40210-630 Salvador, BA, Brazil

**Keywords:** solution blow spinning, heterogeneous structures, bioactive wound dressing, ibuprofen, drug delivery

## Abstract

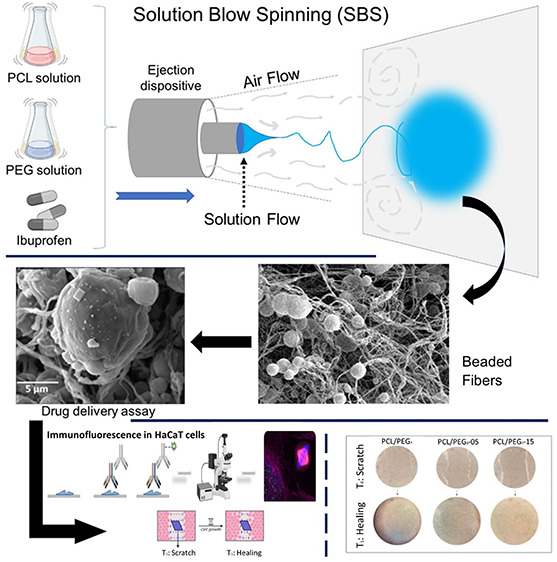

The regeneration of skin injuries can be aided by tissue engineering strategies, which enable the recovery of the structural and functional integrity of the damaged tissue. The Solution Blow Spinning (SBS) technique has attracted the attention of researchers due to the production of nanofiber mats in a continuous process, which exhibit high porosity and the ability to deliver drugs locally. The objective of this work was to produce and encapsulate ibuprofen in mats of PCL/PEG as a fast-acting analgesic drug delivery system. Initially, beaded nanofiber structures were produced from PCL solutions in chloroform at 8% (w/v) and PCL/PEG solutions in mass ratios of 2:1 and 1:1. The influence of the PEG concentration, gas pressure (compressed air), and solution injection rate on the fibers’ morphology was analyzed by SEM. Then, the best condition for the formation of PCL/PEG beaded fiber structure was selected (1:1, 137.90 kPa, and 7.2 mL/h) for the fabrication of the mat containing ibuprofen at proportions of 5, 15, and 30% by polymer mass (PCL/PEG). The SBS-spun mats demonstrated a remarkable swelling capacity of approximately 400%, with bead presence enabling a gradual release of ibuprofen within the first 5 h. Additionally, the wound-healing assay confirmed that ibuprofen-loaded PCL/PEG_8_ mats significantly promoted NF migration, suggesting their potential to accelerate the wound-healing process.

## Introduction

Wound dressings play a crucial role in promoting healing by providing physical protection, maintaining a moist environment, and enabling controlled drug delivery. Advanced dressings, such as nanofibrous scaffolds, mimic the extracellular matrix, thereby enhancing cell migration and proliferation. Conventional dressings, on the other hand, present significant limitations, including rapid drug release (burst effect), inadequate exudate absorption, and high production costs. These challenges underscore the need for innovative materials that integrate both structural and functional properties to effectively address the complexities of chronic wound healing.^1−4^

Among recent innovations, thin and transparent films enable the visual monitoring of the healing process, while nanofibrous dressings mimic the skin extracellular matrix (ECM), facilitating improved cell migration and proliferation.^5^ However, these advanced dressings present challenges, including the rapid release of encapsulated drugs, which limits their long-term efficacy.^6,7^

Filler dressings are essential in managing chronic wounds, particularly those with significant tissue loss.^8,9^ These materials are specifically designed to fill the wound bed, absorb exudates, maintain a moist environment, and promote tissue regeneration.^10^ The integration of structural and functional properties in hybrid dressings composed of nanofibers and microbeads (beaded fibers) has demonstrated great potential for addressing these challenges.^10−13^

Techniques such as solution blow spinning (SBS) have been investigated as a way to overcome production and efficiency challenges in wound dressings.^14^ This technology facilitates the fabrication of hybrid fibrous matrices capable of combining both rapid and sustained drug release. Polymers such as poly(ε-caprolactone) (PCL) and poly(ethylene glycol) (PEG) are commonly used due to their biocompatibility, biodegradability, and tunable properties, including improved hydrophilicity and absorption capacity. These attributes are critical for the development of filler dressings specifically designed for chronic wounds.^15^

Ibuprofen, a widely used nonsteroidal anti-inflammatory drug (NSAID), is frequently incorporated into topical wound dressings due to its dual potential to promote healing and alleviate pain in chronic wounds. Recent studies have demonstrated that integrating NSAIDs into transdermal drug delivery systems offers an effective way to deliver these agents directly to the wound site, thereby reducing inflammation and providing targeted pain relief.^16−20^

In addition to its anti-inflammatory and analgesic properties, ibuprofen has been shown to reduce scar formation, further highlighting its potential as a promising drug for wound healing.^21−23^ Notably, wound temperature during the healing process typically ranges from 30 to 34 °C, with elevated temperatures being linked to impaired healing. This has led to interest in the development of thermoresponsive drug delivery systems, such as those incorporating ibuprofen, as a significant innovation in wound care. These systems function as temperature-sensitive carriers, releasing drugs only when the wound temperature exceeds the normal range, thereby enabling targeted and controlled delivery.^24,25^

Polycaprolactone (PCL) is a synthetic polyester extensively used in the production of wound dressings due to its biocompatibility, biodegradability, and noncytotoxicity, and is approved by the Food and Drug Administration (FDA). However, its hydrophobic nature presents a limitation, reducing its capacity to absorb exudates and effectively regulate wound environments.^26,27^

Polyethylene glycol (PEG) is a hydrophilic, nontoxic, and biocompatible polymer with high swelling capacity, also approved by the FDA. It is synthesized through the polymerization of ethylene oxide.^28^ Blending PCL with PEG can improve its biodegradability and hydrophilicity, making the composite more suitable for short-term drug delivery systems.^29^

Although previous studies have investigated nanostructured matrices produced via electrospinning as a drug encapsulation platform for nonsteroidal anti-inflammatory drugs (NSAIDs) such as ibuprofen,^30,31^ limited research has focused on the use of the solution blow spinning (SBS) technique to produce PCL/PEG mats with mixed structures of fibers and beads.

The objective of this study was to produce mats with beaded nanofiber structures of PCL/PEG using the solution blow spinning (SBS) technique, incorporating different concentrations of ibuprofen for potential application in wound dressings. To achieve this, the spinnability of PCL/PEG blends via SBS was assessed through morphological analysis. The relationship between PCL (8% wt/v) solutions and PEG was studied using mass ratios of 2:1 and 1:1. The spinning process was further optimized by varying gas pressure and injection rate parameters.

The chemical composition of beaded-fiber mats was analyzed both before and after ibuprofen release to determine the presence of PEG within the spun mats. Drug release behavior in saline solution was evaluated using UV–vis spectroscopy.

## Materials and Methods

### Materials

Polycaprolactone (PCL) (*M*_w_ = 93,416 g/mol, *M*_n_ = 59,920 g/mol) was obtained from Perstorp (United Kingdom) in the form of pellets. Polyethylene glycol (PEG) (*M*_n_ = 4000 g/mol) was purchased from Vetec Quimica Fina (São Paulo, Brazil). Ibuprofen (IBU) (fine white powder with 99.95% purity and 97.5% anhydrous base content), used as the model drug, was fabricated in Alka Laboratories (India), but purchased from Delaware Importadora Química (lot 336/21, Brazil). Chloroform was obtained from Sigma-Aldrich, São Paulo, Brazil.

### Preparation of the Beaded-Fiber Mats

The mats were produced by the Solution Blow Spinning apparatus described by Carlos et al.^32^ Polymeric solutions were prepared using chloroform as a solvent. One solution consisted of PCL at 8 wt %, while two separate PEG solutions were prepared at 8 and 4 wt %. To obtain the final PCL/PEG solutions, the same volume of the PCL solution (8 wt %) was mixed with the PEG solution. More specifically, PCL/PEG_4_ refers to the mixture of PCL (8 wt %) and PEG (4 wt %), resulting in a 2:1 mass ratio (PCL:PEG), while PCL/PEG_8_ corresponds to the 1:1 mass ratio, obtained by mixing equal concentrations of PCL (8 wt %) and PEG (8 wt %). The solution containing ibuprofen was prepared by dissolving ibuprofen at 5, 15, and 30 wt % of the dry weight of blended polymeric in the PCL/PEG (1:1) solution. The concentration of the solutions used is displayed in Table 1. The mats produced from solutions containing ibuprofen are referred to as PCL/PEG_8_-IBU throughout the text.

**Table 1:** Samples and the Respective Composition of the Solutions Used To Spin Them^a^

sample	PCL (wt %)	PEG (wt %)	IBU (wt % PCL/PEG)
PCL	8		
PCL/PEG_4_	8	4	
PCL/PEG_8_	8	8	
PCL/PEG_8_-5	8	8	5
PCL/PEG_8_-15	8	8	15
PCL/PEG_8_-30	8	8	30

a All solutions were prepared with chloroform as solvent.

### Viscosimeter

The viscosity of PCL/PEG solutions shown in Table 1 was evaluated using a rotational viscometer (Anton Paar) with spindle CC18, at room temperature (∼25 °C). The shear flow was varied at five points (12 to 300 s^–1^), maintaining a range of 10–80% of deformation rate.

### Morphological Evaluation of Spun Mats

The morphologies of the PCL and PCL/PEG mats were analyzed using scanning electron microscopy (Versa 3D Dual Beam-FEI) with an acceleration of 10 kV. The samples were coated with gold for 120 s. The diameters of fibers and beads were determined using ImageJ software. For this, the diameters of 50 fibers and 30 beads per film were measured.

### Fourier-Transform Infrared Spectroscopy (FTIR)

A Fourier-transform infrared spectrometer equipped with an attenuated total reflectance accessory (ATR-FTIR) and a ZnSe crystal (Nicolet, model 6000–Thermo Scientific) was used to assess the chemical composition and the mats to evaluate the PCL/PEG interaction within them. Spectra were obtained in the range of 4000 to 650 cm^–1^ with 128 scans per sample, with a resolution of 4 cm^–1^ in transmittance mode.

### Thermogravimetric Analysis (TGA)

The thermal stability of PCL/PEG-Ibuprofen samples and ibuprofen powder was studied by thermogravimetric analysis (TGA), using a TGA-50 Shimadzu apparatus. The analysis was conducted under a nitrogen atmosphere, from 25 to 600 °C, with a heating rate of 10 °C/min. The ibuprofen content in PCL/PEG mats was estimated from TGA curves (*n* = 5).

### Differential Scanning Calorimetry (DSC)

The thermal behavior of PCL/PEG_8_ and PCL/PEG_8_-IBU mats was evaluated by differential scanning calorimetry (DSC) using a Hitachi High-Tech DSC 7000 series (Japan). In this analysis, 10 mg per sample was subjected to two heating cycles and one cooling cycle, with a heating rate of 10 °C/min under a nitrogen atmosphere with a flow rate of 50 mL/min. The first heating cycle was conducted from −20 to 100 °C, followed by the cooling cycle to −80 °C, and subsequently heated to 100 °C. Equation 1 was used to calculate the crystallinity degree of the blends. The data were processed using the second heating cycle of DSC in OriginPro 2024b software.
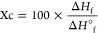
1where Δ*H*_f_ is the melting enthalpy of the endothermic peak of the second heating of the DSC thermogram, while Δ*H*_f_° = 151.7 J/g is the theoretical melting enthalpy for a 100% crystalline PCL sample.^33^

### Swelling Assay

The water uptake capacity of the PCL/PEG_8_-IBU mats was evaluated by immersing 2 cm × 2 cm mats in 20 mL of saline solution (0.9 wt % NaCl) at 37 °C. The mats were removed from the Falcon tubes, excess liquid was blotted off, and they were weighed after 45 min, 2, 3, 4, 5, 24, and 48 h of immersion. The swelling ratios of the films (%) were calculated based on eq 2.
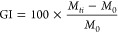
2where GI is the swelling rate, *M*_0_ is the initial mass of the mat, and *M_ti_* is the mass of the mat at the specified immersion time.

### In Vitro Ibuprofen Release Studies

The drug release studies of the PCL/PEG_8_-IBU samples were evaluated by soaking 2 cm × 2 cm mats in 20 mL of saline solution (0.9 wt % NaCl) at 37 °C. At time intervals of 0.5, 1, 2, 3, 4, 5, 24, and 48 h, 2 mL of the solution was withdrawn and replaced with 2 mL of fresh saline solution. All absorbance measurements were conducted using a PerkinElmer Lambda 25 UV-Vs spectrophotometer (Boston, USA). The addition of fresh saline solution to the Falcon tube altered the IBU concentration; Therefore, a correction factor was applied, as described in eq 3.
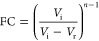
3where FC is the correction factor, *V*_i_ is the volume for the release medium, *V*_r_ is the withdrawn volume, and *n* is the number of withdrawn times.^34^

### In Vitro Biological Assays of Spun Mats

#### Cell Lines

Different cell lines were used in this study to analyze the impact of the treatment on nontransformed cell lines: Human umbilical vein endothelial cells (HUVEC) were kindly provided by Pilar Sánchez Gómez, Normal (Nontumor-associated) fibroblasts (NF) were kindly donated by Akira Orimo; these cells were obtained from a woman undergoing reduction mammoplasty.^35^ The spontaneously transformed keratinocyte cell line (HaCaT) was kindly donated by Penny Lovat. All cell lines were cultured in DMEM and supplemented with 10 wt % fetal bovine serum and 1% penicillin/streptomycin and maintained under standard culture conditions (37 °C in a humidified atmosphere with 5% CO_2_).

#### Cell Viability Assay

For viability assays, cells were seeded on the surface of the PCL/PEG_8_ and PCL/PEG_8_-IBU mats at a density of 2 × 10^3^ cells/cm^2^ per well in 6-well plates and incubated in complete medium in the presence of different stimuli for 48 h. Then, they were subjected to the MTT assay, a colorimetric test used to measure cell metabolic activity and estimate cell viability.

The MTT solution (0.5 mg/mL) was added to each well in a 1:10 dilution. Cells were subsequently incubated for 3 h under standard cell culture conditions to allow the formation of the formazan crystals. Isopropyl alcohol (1:1) was then added to each well to dissolve the formazan crystals, and absorbance was subsequently measured at 570 nm using a SpectraMax M3 microplate reader.

All experiments were performed in triplicate, and the results were expressed as the percentage of viable cells compared to the control group (only cells). The negative control condition consisted of cells not incubated with the PCL/PEG_8_ mats.

### Non-Contact Coculture of NF and PCL/PEG Mats: Cell Viability Assay

To analyze cell viability while avoiding the direct contact between PCL/PEG_8_ and PCL/PEG_8_-IBU samples with the cells, cell viability assays were performed using Boyden chambers (#734-2748; VWR). These chambers consist of two reservoirs separated by a polyethylene terephthalate (PET) porous membrane with an 8 μm pore size.

The PCL/PEG_8_-loaded mats were placed in the upper reservoir with DMEM medium, ensuring physically separation from the lower reservoir, where fibroblasts were cultured in DMEM supplemented with 10 wt % fetal bovine serum and 1% penicillin/streptomycin.

To perform the tests, the Boyden chambers were placed inside the wells of a 24-well plate, at the bottom of which the NFs were seeded at a density of 1 × 10^3^ cells/cm^2^ per well and incubated for 48 h with the mats. Cell viability was determined using the MTT assay.

### Wound Healing Assay

To perform this experiment, approximately 5 × 10^3^ cells/cm^2^ (NFs) in complete DMEM medium were seeded in a 6-well plate for 24 h. Then, the cell monolayer was scratched with a yellow pipette tip, and the PCL/PEG_8_ and PCL/PEG_8_-IBU mats were inserted in each well.

**Figure 1:**
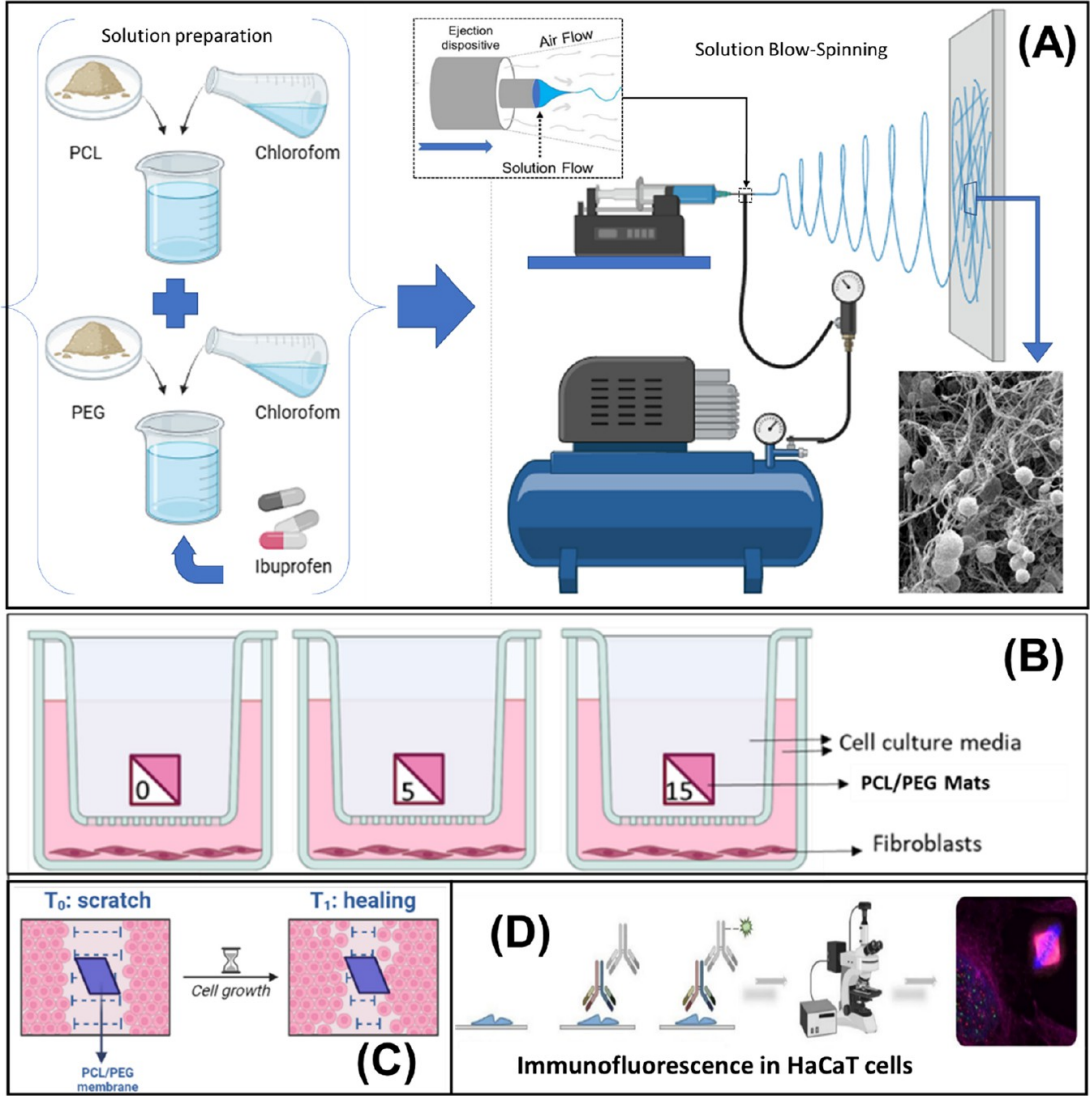
Illustration images of fast-acting analgesic drug delivery system preparation (A), and different in vitro assays: (B) noncontact coculture of ibuprofen-loaded PCL/PEG_8_ mats with NF; (C) wound healing assay scheme. (D) Immunofluorescence scheme in HaCaT cells.

Cell migration into the wounded area was observed under an inverted microscope after 24 h, and images were captured at the indicated time points. Measurements were taken from five individual microscopic fields in each experiment. The migration area was determined by measuring the total area of the wound using the ImageJ software, as shown in Figure 1B.

### Western Blot

Western blot analysis was performed following standard procedures (Figure 1C). Briefly, proteins were extracted using RIPA buffer [150 mM NaCl, 1% (v/v) NP40, 50 mM Tris-HCl pH 8.0, 0.1% (v/v) SDS, 1 mM EDTA, 0.5% (w/v) deoxycholate]. Total protein was quantified by using the Bradford method, dissolved by SDS-PAGE on 12% acrylamide gels (Bio-Rad, Hercules, CA, USA), transferred to polyvinylidene difluoride (PVDF) membranes, blocked in a 5% skim milk solution or 5% BSA (Sigma) and incubated at 4 °C overnight with a primary antibody (see below the list of the primary antibodies used in this study). After washing, the membranes were subsequently incubated with the corresponding horseradish peroxidase (HRP)-conjugated antimouse or antirabbit secondary antibodies (1:5000; GE Healthcare, Chicago, IL, USA) and visualized by enhanced chemiluminescence (Bio-Rad, Hercules, CA, USA). The images were obtained with the ImageQuant LAS 500 chemiluminescence CCD camera (GE Healthcare Life Sciences, Chicago, IL, USA). ECL results were scanned, and the amount of each protein band was quantified using NIH ImageJ software (NIH Image, Bethesda, MD, USA, http://rsb.info.nih.gov/nih-image/).

The following primary antibodies were used in this study: anti-LC3 (1:2000; Sigma-Aldrich, #L7543, St. Louis, MO, USA), anticleaved PARP Asp214 (1:1000; Cell Signaling, #9541), antitubulin (1:5000; Sigma, T9026).

### Immunofluorescence Straining

HaCaT cells were plated onto glass coverslips, fixed with 4% paraformaldehyde (Sigma) for 10 min, permeabilized, and blocked with 10% goat serum PBS containing 0.25% Triton X-100 (Sigma) for 1 h and incubated with the corresponding primary antibodies anti-LC3 (1:200, Sigma, L754, St. Louis, MO, USA) and anticleaved Caspase3 (1:200, Abcam, ab32042, UK) in 5% goat serum overnight at 4 °C. Coverslips were washed with PBS and incubated with the corresponding antirabbit Alexa-488-conjugated secondary antibodies (Life Technologies, Carlsbad, CA, USA) at a 1:1000 dilution at room temperature for 1 h and nuclei were stained with DAPI (Roche) for 10 min. Finally, coverslips were mounted with mowiol (Calbiochem-Merck), and images were obtained using a Leica TCS SP2 confocal microscope (Leica, Germany). Measurements were taken from three individual microscopic fields in each experiment. The representative data from each experiment are presented in Figure 1D.

## Results and Discussion

Studies on drug release from nanofibers have predominantly reported a “burst″ release, which poses a drawback for controlled release systems utilizing these morphologies. This phenomenon is primarily attributed to the high surface area of nanostructured fibrous mats, which accelerates polymer erosion and facilitates drug diffusion through the encapsulating polymeric material.^36^ To mitigate the burst effect, beaded-fiber structures have been investigated and applied, leveraging microbeads as potential drug reservoirs, while nanofibers provide structural support, enhancing the handling and applicability of the mats as wound dressings for the skin.

### Solution Viscosity Evaluation

**Figure 2:**
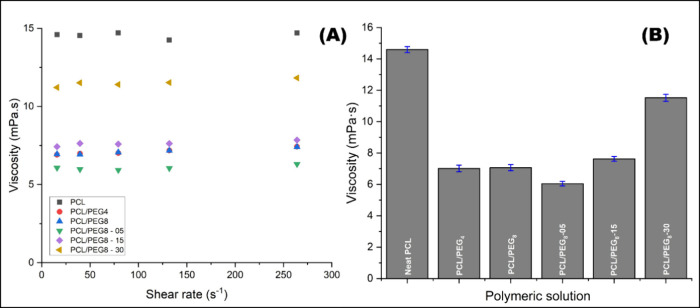
Rheological behavior (A) and viscosity (B) of PCL/PEG solution with and without ibuprofen.

One of the most significant variables affecting the morphology of structures spun via solution blow spinning (SBS) is the viscosity of the polymeric solutions, as it is directly related to the degree of entanglement and interaction of the polymer–solvent chains. This viscosity determines whether the solution will form fibers in more viscous conditions or microspheres in lower-viscosity and more diluted regimes.^37^ Based on this, a rheological behavior study was conducted (Figure 2) for PCL/PEG solutions with varying ibuprofen concentrations.

Figure 2 shows the Newtonian behavior of the PCL and PCL/PEG solutions, both with and without ibuprofen. The absolute viscosity did not exhibit significant variations in any of the solutions when subjected to different shear rates.

On the other hand, Figure 2B illustrates the viscosity decrease of polymeric solutions from ∼14.5 mPa·s for pure PCL (neat PCL) to 7.02 and 7.07 mPa·s for solutions with PCL/PEG mass ratios of 2:1 (PCL/PEG_4_) and 1:1 (PCL/PEG_8_), respectively. This result indicates that the incorporation of PEG did not significantly influence viscosity, as variations in PEG content did not lead to substantial changes in viscosity.

However, these solutions exhibited an approximate 50% viscosity reduction compared to the pure PCL solution, which may be attributed to the dilution effect of the initial PCL solution when mixed with the PEG solution, as well as the lubricating effect between PCL chains.^38,39^

On the other hand, the incorporation of ibuprofen into PCL/PEG_8_ (1:1) solutions led to significant changes in viscosity. At a 5 wt % ibuprofen concentration (PCL/PEG_8_-05), there was an approximately 10% (PCL/PEG_8_-15) reduction in viscosity compared to solutions without ibuprofen (PCL/PEG_8_). This reduction may be attributed to the lubricating effect of ibuprofen within the polymeric chains, facilitating their movement in the solution and thereby decreasing viscosity.^40^

Conversely, solutions with a higher ibuprofen content (15 and 30 wt %) exhibited an increase in viscosity (Figure 2), which may be attributed to the possible emulsification of the solutions induced by ibuprofen, creating stronger interactions between the polymeric chains and the drug molecules.^34^

### Beaded-Fiber Morphology Evaluation

PCL is a widely used polymer in the production of nanostructured matrices via solution blow spinning for wound dressing applications. However, these mats exhibit low moisture absorption and retention, which is a crucial characteristic for skin injury regeneration, particularly in burns.^41^ Therefore, the first phase of this study focused on the incorporation of PEG, a hygroscopic polymer that improves the wettability of wound dressings.^42^

**Figure 3:**
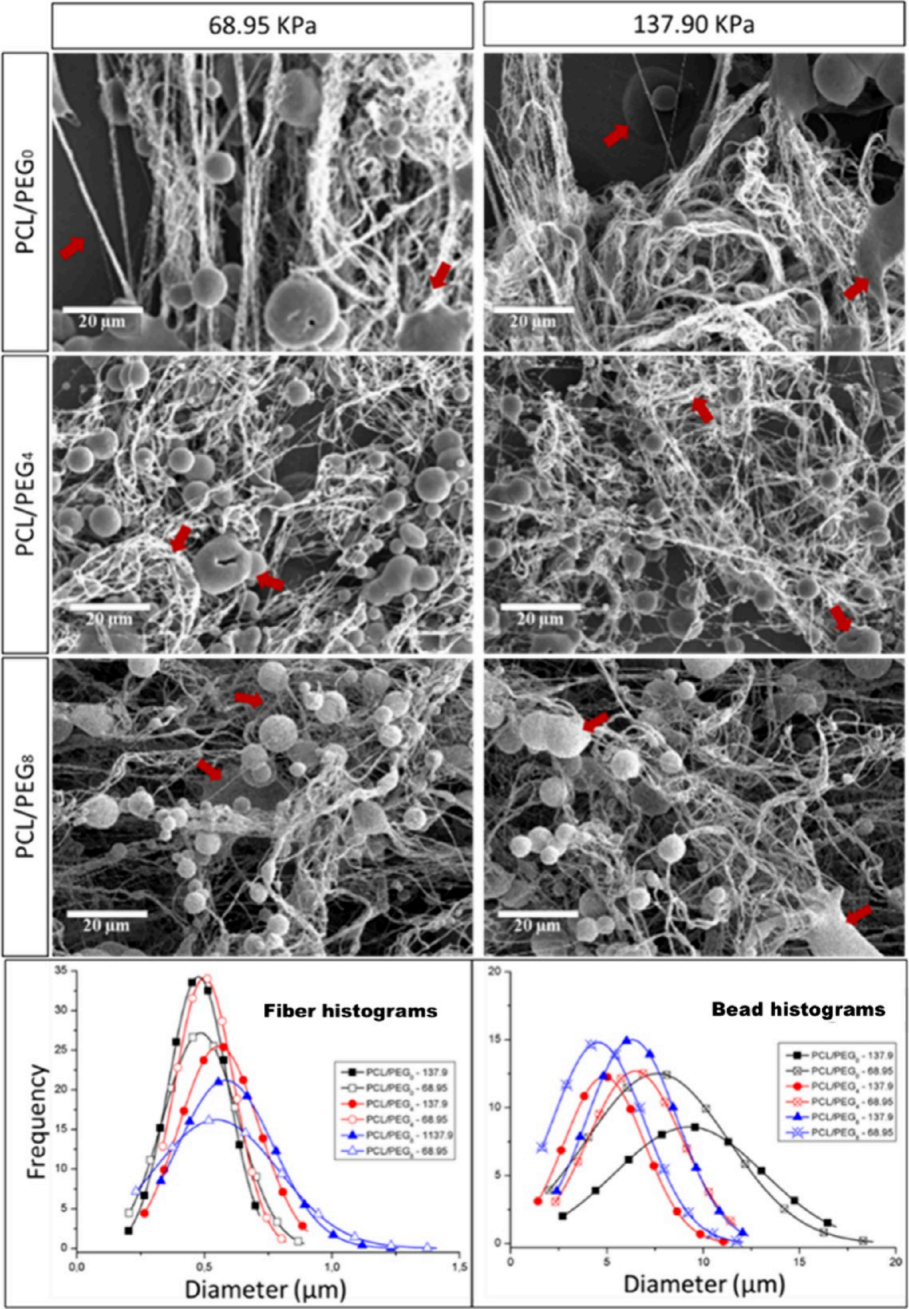
SEM images showing the influence of the gas pressure and the composition of PEG on the morphology at a fixed injection rate of 6.0 mL/h.

The study evaluated different mass ratios between PCL and PEG, with a fixed PCL concentration of 8 wt % and varying PEG concentrations at 0, 4, and 8 wt % (mass ratios of 1:0, 2:1, and 1:1), referred to as PCL/PEG_0_, PCL/PEG_4_, and PCL/PEG_8_, respectively. Gas pressure was varied at 68.95 and 137.90 kPa for the production of these mats, while other variables, such as tip needle tip-collector distance kept constant (at 30 cm) and flow rate, were studied with variations at two points: 6.0 and 7.2 mL/h. Figure 3 shows the effect of gas pressure and polymer concentration on the heterogeneous morphologies, as evaluated by SEM images with the flow rate maintained at 6.0 mL/h.

Figure 3 shows the morphology of heterogeneous structures for different mats spun via SBS, varying the applied air pressure and the mass ratio between PCL and PEG. As shown, for all evaluated air pressure and PEG concentration conditions, the matrices exhibited hybrid structures composed of nanofibers and microbeads, characteristic of the beaded-fiber structure.^43,44^ However, measurements of the evaluated structures (fibers and beads) did not show significant variations, as illustrated in the histograms in Figure 3. Furthermore, the results obtained in this study align with those reported by Li et al.,^45^ who evaluated the production of heterogeneous structures and demonstrated that air pressure was not a major factor influencing the fibrillar morphology of the studied structures, as the fiber diameters exhibited very similar values for the two applied pressures. This is consistent with the findings in the literature, which indicate that the solution concentration is the most influential variable in SBS-produced structures.^32,46^

In summary, the incorporation of PEG into the PCL solution for SBS resulted in the formation of heterogeneous morphologies with fewer defects (Figure 3; red arrows).^43^ However, in SBS, the addition of PEG led to an increase in the standard deviation of fiber diameter and a decrease in bead diameter, indicating a reduction in solution viscosity and greater instability during jetting.^47^

This effect may be attributed to the lubricating action of PEG, due to its low molecular weight, which reduces polymer–polymer interactions and promotes the formation of more uniform droplets, making the technique more similar to Solution Blow Spraying.^48^

The increase in bead formation is also evident in the data from Table 2. These findings align with previous studies indicating that the solution concentration is the most influential variable in SBS-produced structures. This reduction in bead formation at higher air pressures is attributed to increased aerodynamic forces, where turbulence causes great material loss compared to mats produced at lower air pressures. This phenomenon has been extensively studied in the literature, and the influence of air pressure and velocity on the morphology of electrospun fibers has been reported by various authors.^49,50^

**Table 2:** Fiber/Bead Diameter Ratio as a Function of the Ibuprofen Amount Incorporated in the Mat Evaluated

sample	fiber diameters [nm]	bead diameters [μm]	amount of beads by area evaluated^a^
PCL/PEG_8_	(5.1 ± 1.5) × 10^2^	3.9 ± 2.5	214
PCL/PEG_8_-05	(5.1 ± 2.6) × 10^2^	6.4 ± 3.0	135
PCL/PEG_8_-15	(5.2 ± 2.2) × 10^2^	5.7 ± 2.1	125
PCL/PEG_8_-30	(6.3 ± 3.4) × 10^2^	5.1 ± 2.9	64

a Area: 208 × 150 μm^2^.

**Figure 4:**
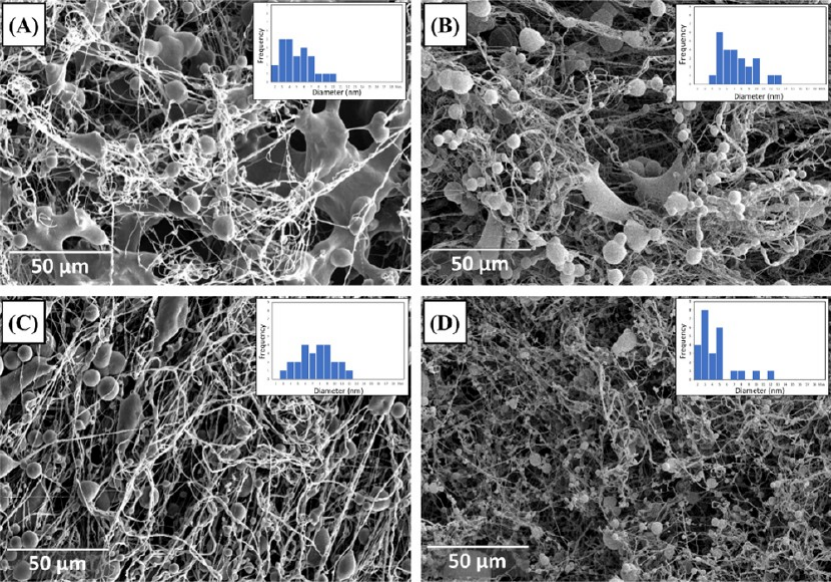
SEM images and bead histogram for the PCL/PEG_8_ beaded nanofibers spun with flow rate variation and the influence on the morphology of the two air pressures used. Flow rate: 6.0 mL/h, (A) 68.95 kPa; (B) 138.90 kPa, and flow rate: 7.2 mL/h (C) 68.95 kPa; (D) 138.90 kPa.

To evaluate the influence of flow rate on the spinning process and the formation of beaded-fiber structures via SBS, a second flow rate (7.2 mL/h) was studied. Figure 4 illustrates the morphological variations of the structures compared to those obtained at a flow rate of 6.0 mL/h (Figure 3).

In Figure 4, greater homogeneity of the structures obtained with an increased flow rate is evident. Figure 4A,B shows the morphology of the beaded-fiber structures produced at a flow rate of 6.0 mL/h, highlighting challenges in chain stretching and solvent evaporation. These issues are attributed to the presence of dense plate-like structures in both conditions.

In contrast, these defects were not observed in samples spun at a flow rate of 7.2 mL/h under an air pressure of 68.95 kPa (Figure 4C). These samples exhibited a higher quantity of fibers with an average diameter of 643 ± 199 nm and beads measuring 703 ± 245 μm. At a higher air pressure of 138.9 kPa (Figure 4D), a significant reduction in the diameter of both fibers and beads was observed, measuring 519 ± 155 nm and 393 ± 256 μm, respectively.

This improvement can be primarily attributed to the stronger stretching force exerted on the polymer solutions, which facilitates molecular reorganization and leads to more homogeneous structures compared to those obtained at lower flow rates under the air pressures used in this study.

Finally, the processing conditions chosen to continue this study focused on the morphology of the beaded fibers and the distribution of fiber and bead diameters. Thus, the sample produced using the PCL/PEG_8_ solution with 7.2 mL/h and a pressure of 137.9 kPa, produced nanofibers and microbeads with more homogeneous diameters. For this reason, these conditions were chosen for the ibuprofen encapsulation study.

### Beaded-Fiber Morphology of PCL/PEG with Ibuprofen

**Figure 5:**
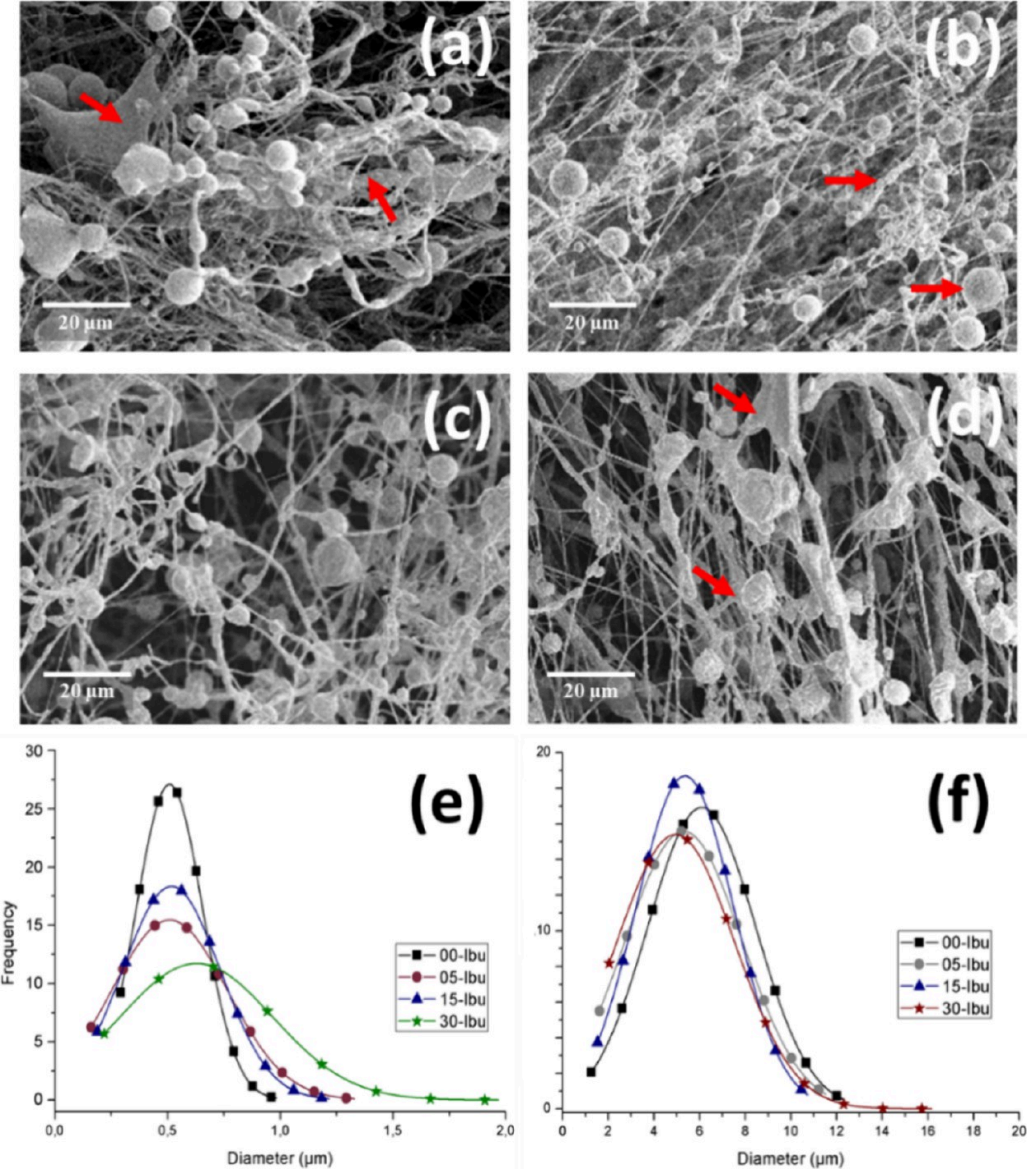
SEM Images of PCL/PEG_8_ beaded-fibers varying the ibuprofen concentration: 0 wt % (a) PCL/PEG_8_, 5 wt % (b) PCL/PEG_8_-05, 15 wt % (c) PCL/PEG_8_-15, and 30 wt % (d) PCL/PEG_8_-30, and histograms for (e) fibers and (f) beads. (Flow rate: 7.2 mL/h and air flow: 137.9 kPa).

With the selected process configuration, PCL/PEG beaded-fibers carrying varying concentrations of ibuprofen were produced in mat form. Figure 5 illustrates the morphology of these structures, while Table 2 presents the average diameters of the structures and the number of beads obtained in this study as a function of ibuprofen (Ibu) concentration.

In Figure 5, the influence of the ibuprofen concentration on the beaded-fiber morphology is evident as the fiber diameters visibly increase with higher drug concentrations. Additionally, the presence of the drug appears to affect the concentration of defects, leading to an increase in their number as the concentration of ibuprofen rises.

This also impacts the shape of the defects, which can be more easily observed in Figure 5d, where nonspherical defects with a “slab-like” form are predominantly seen.^43^ These images reveal a significant presence of junctions and the formation of less homogeneous fibers as the drug concentration increases.

A key challenge in processing nonhomogeneous materials is ensuring reproducibility, as variations in structural morphology can significantly impact the properties of the final material. In solution blow-spinning (SBS), the formation of beads and fibers is influenced by the polymer solution properties, solvent evaporation rate, and processing parameters such as airflow and polymer feed rate.^32^ To evaluate these variations, Table 2 presents the fiber and bead diameters, as well as the bead count per evaluated area, for different formulations incorporating ibuprofen.

The results indicate that ibuprofen incorporation affects both the bead diameter and the number of beads per unit area. In the PCL/PEG_8_ control sample, bead diameters averaged 3.9 ± 2.5 μm, with 214 beads per analyzed area. As the ibuprofen concentration increased to 5.0 wt % (PCL/PEG_8_-05), the beads became larger (6.4 ± 3.0 μm) while their total number decreased (135 beads per area). A similar trend was observed for 15 (PCL/PEG_8_-15) and 30 wt % (PCL/PEG_8_-30), where bead diameters fluctuated, and their concentration was further reduced (125 and 64 beads per area, respectively).

This behavior can be attributed to changes in the solution viscosity and solvent evaporation dynamics caused by the incorporation of ibuprofen. Higher drug concentrations probably affect polymer chain entanglements and jet breakup during fiber formation, leading to larger beads and a reduction in their occurrence. Additionally, the solvent evaporation rate and polymer–air interactions play a crucial role in bead formation in SBS, where rapid solvent removal can lead to fiber stretching, whereas slower evaporation may favor bead formation.^26^

Although minor variations in bead size and distribution were observed across different runs, the overall trend remained consistent, demonstrating an acceptable level of reproducibility. These findings highlight the importance of controlling solution composition and processing conditions to optimize the morphology of SBS-produced fibers for drug delivery applications.

### Mats Characterization

#### Composition Evaluation by FTIR

The chemical composition of the PCL/PEG_8_ mats, as well as the incorporation of ibuprofen, were evaluated using FTIR-ATR.

**Figure 6:**
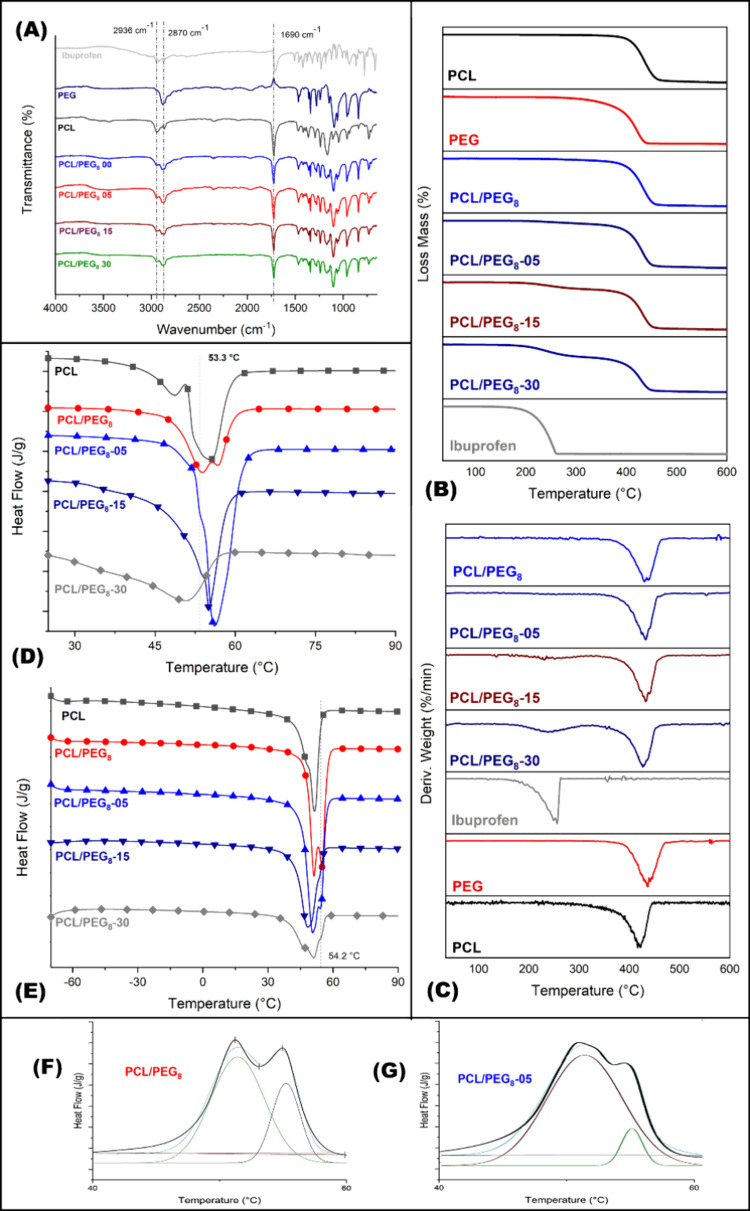
Chemical and thermal characterization of PCL/PEG_8_ mats with different amounts of ibuprofen loaded. (A) FTIR-ATR spectra; thermogram of spun samples and ibuprofen powder (B) TGA, and (C) DTG. Thermal transition by DSC of blend PCL/PEG beaded-nanofibers (D) first heat cycle, (E) second heat cycle, and deconvolution analysis of each melt peak: (F) PCL/PEG_8_, and (G) PCL/PEG_8_-05 mats.

Figure 6A shows the characteristic bands for PCL and PEG, with a band observed at 2963 cm^–1^ related to the stretching of the methylene (CH) group, due to the infrared radiation absorption of the C–H groups attached to the carbon side chains of PCL. Additionally, a characteristic band at 1690 cm^–1^, corresponding to the stretching vibrations of the carbonyl group (C=O), and aromatic C=C stretching at 1502 cm^–1^, present in esters, were retained in the physical mixture.^51,52^

Regarding the characteristic bands of PEG, a prominent band at 2870 cm^–1^, related to the symmetric stretching vibrations (νasCH_2_) of the methylene groups, was observed, attributed to the asymmetry of the C–H bonds in the PEG chain.^53^ However, a decrease in intensity of the 1690 cm^–1^ band (C=O) of PCL was observed with the incorporation of PEG, indicating a low interaction between the polymers. This has previously been reported in the literature for this blend.^54,55^

The peaks present in the ibuprofen spectrum at 2955, 1721, and 1240 cm^–1^ are assigned to asymmetric CH_3_ stretching, C=O stretching and C–O stretching, respectively (Figure 6), while the peaks at 668 and 580 cm^–1^ are related to the aromatic ring vibration in the ibuprofen structure.^56^

However, these ibuprofen bands could not be identified in heterogeneous structures of PCL/PEG_8_ loaded with ibuprofen, due to the overlapping of ibuprofen bands with the polymeric blend.^57,58^

#### Thermal Analysis

Figure 6B shows the thermogravimetric analysis (TGA) performed on PCL/PEG_8_ mats with and without ibuprofen, produced using beaded-fiber mats. The objective was to evaluate the thermal behavior of the samples when three different ibuprofen compositions were incorporated as an active material.

Considering the PCL/PEG_8_ curve, it is possible to observe that the degradation of the structures occurs in a single step, starting at 386 °C and reaching maximum degradation at 430 °C, due to the overlap of the degradation processes of PCL and PEG. Similar results were reported by Ramaswamy et al.,^58^ who analyzed the degradation processes of PCL, PEG, and electrospun PCL/PEG fibers, observing a significant mass loss at 380 °C for the samples.

Through the analysis of the TGA curve for the PCL/PEG_8_ sample, it was possible to observe that no significant mass loss occurred at temperatures below 350 °C, suggesting that the solvent used and the residual moisture were completely evaporated during the spinning process via SBS.

Based on the pure ibuprofen curve, we can observe that the drug’s degradation also occurs in a single step, starting at 202 °C and reaching maximum degradation at 243 °C, presenting a distinct degradation process from the PCL and PEG mass loss processes.

This degradation profile is consistent with the findings of Phaechamud et al.,^59^ who studied the thermal stability of ibuprofen and reported a thermal degradation process starting at 205 °C and reaching maximum degradation at 248 °C. Therefore, it was possible to evaluate the ibuprofen content in the fibers by analyzing the mass loss relative to the drug’s degradation process within the fibers.

Table 3 lists the ibuprofen contents in the mats, as determined through the analysis of TGA curves. The mass loss values were obtained considering the drug degradation process within the approximate range of 180 to 320 °C.

**Table 3:** Encapsulated Ibuprofen Theory in PCL/PEG_8_-IBU Beaded Fibers

sample	ibuprofen theoretical (%)	ibuprofen calculated by TGA (%)	relative error (%)
PCL/PEG_8_-05	4.8	7.6 ± 1.41	58
PCL/PEG_8_-15	13.0	14.3 ± 1.31	10
PCL/PEG_8_-30	23.1	26.2 ± 2,51	13

Based on the data obtained (in triplicate, Figures S1 and S2), it can be observed that the mats contain drug levels which are significantly higher than the theoretical values, with relative errors greater than 5% for all different fibers.^60−62^ This discrepancy may indicate heterogeneity within the mats, which can also be observed by the SEM analysis presented in Figure 4.

Figure 6D,E shows the thermal transitions of the heterogeneous structures of the PCL/PEG_8_ blend with and without ibuprofen. The thermal transitions data are recorded in Table 4. This analysis corresponds to the first heating cycle, aiming to observe the influence of processing on the composite and formulation under study.

**Table 4:** Values of Thermal Transitions Studied by DSC for PCL/PEG_8_ Blends Spun via SBS with Variation in Ibuprofen Concentration

sample	first heat cycle		second heat cycle			Xc (%)
	*T*_m_	*H*_m_	*T*_c_	*T*_m_	*H*_m_	
PCL	55.4	123		51.4	117	77.13
PCL/PEG_8_	51.1	116	–9.1	51.3	118	77.79
PCL/PEG_8_-05	56.2	142	–10.6	50.6	141	92.85
PCL/PEG_8_-15	55.1	147	–6.4	48.3	108	
PCL/PEG_8_-30	50.6	144	–9.0	51.0	71.6	

Figure 6D shows the thermal behavior of the material during the SBS processing (first thermal cycle). It can be observed that the spun PCL mats exhibited two characteristic endothermic peaks corresponding to the heat flow required to melt two populations of crystals present in the material. The first peak corresponds to the formation of a smaller population of more imperfect crystals, which required less heat to melt (47.3 °C). This result is consistent with literature reports on PCL spinning.^34,58^

The incorporation of PEG (PCL/PEG_8_) in equal mass proportions resulted in the observation of a second melting peak, corresponding to two populations of crystals similar to those in pure PCL. This second peak (55 °C) could be attributed to the enthalpy of fusion of PEG crystals. Since PCL crystals melt before those of PEG, this behavior suggests incompatibility in the blend, attributed to this crystallization process.

As PCL and PEG have similar melting temperatures (*T*_m_), the crystallinity of neat PCL and its blend with PEG was evaluated by deconvolution of the melting peak obtained during the second heating cycle of DSC (Figure 5E) for the PCL, PCL/PEG_8_, and PCL/PEG_8_-05 samples. This analysis aimed to understand the structural changes in the crystalline phase within the blend and assess how ibuprofen might interfere.

Figure 5F,G shows the DSC thermograms for the melting peak of the PCL/PEG_8_ and PCL/PEG_8_-05 samples, where the presence of two characteristic peaks for PCL and PEG is evident. The area of the deconvoluted PCL peaks revealed that the enthalpy of fusion for PCL in the blend was similar to that of pure PCL, indicating a low interaction between the two polymers used.

For the sample with 5% ibuprofen, an approximately 20% increase in PCL crystallinity was observed (Table 4), suggesting that this amount of drug acted as a nucleating agent for PCL.^10^ This result diverges from that reported by Tri and Prud’homme,^29^ who reported a reduction in PCL crystallinity when blended with PEG, using a PCL of lower molar mass.

As seen in Figure 2, the inclusion of ibuprofen in the blend enhanced polymer interaction, attributed to the significant increase in the enthalpy of fusion for samples with 5 wt % ibuprofen. This quantity of drug acted as a nucleating agent for the PCL/PEG_8_ blend. However, for samples with a higher ibuprofen content (15 and 30 wt %), a decrease in the heat requirements related to the polymer enthalpy of fusion was observed, suggesting that lower crystal formation occurred in the PCL/PEG blend, indicating that ibuprofen acted as a lubricant agent.^55,63^

However, in this study, it cannot be said that a chemical interaction occurred between the two polymers, as described by Kheilnezhad and Hadjizadeh,^55^ since the melting temperature (*T*_m_) of the PLC/PEG_8_ blend crystal showed no variation compared to the *T*_m_ of pure PCL in both heating cycles, as shown in Table 4, indicating little interaction between the polymers and the drug.

#### Swelling Assay

**Figure 7:**
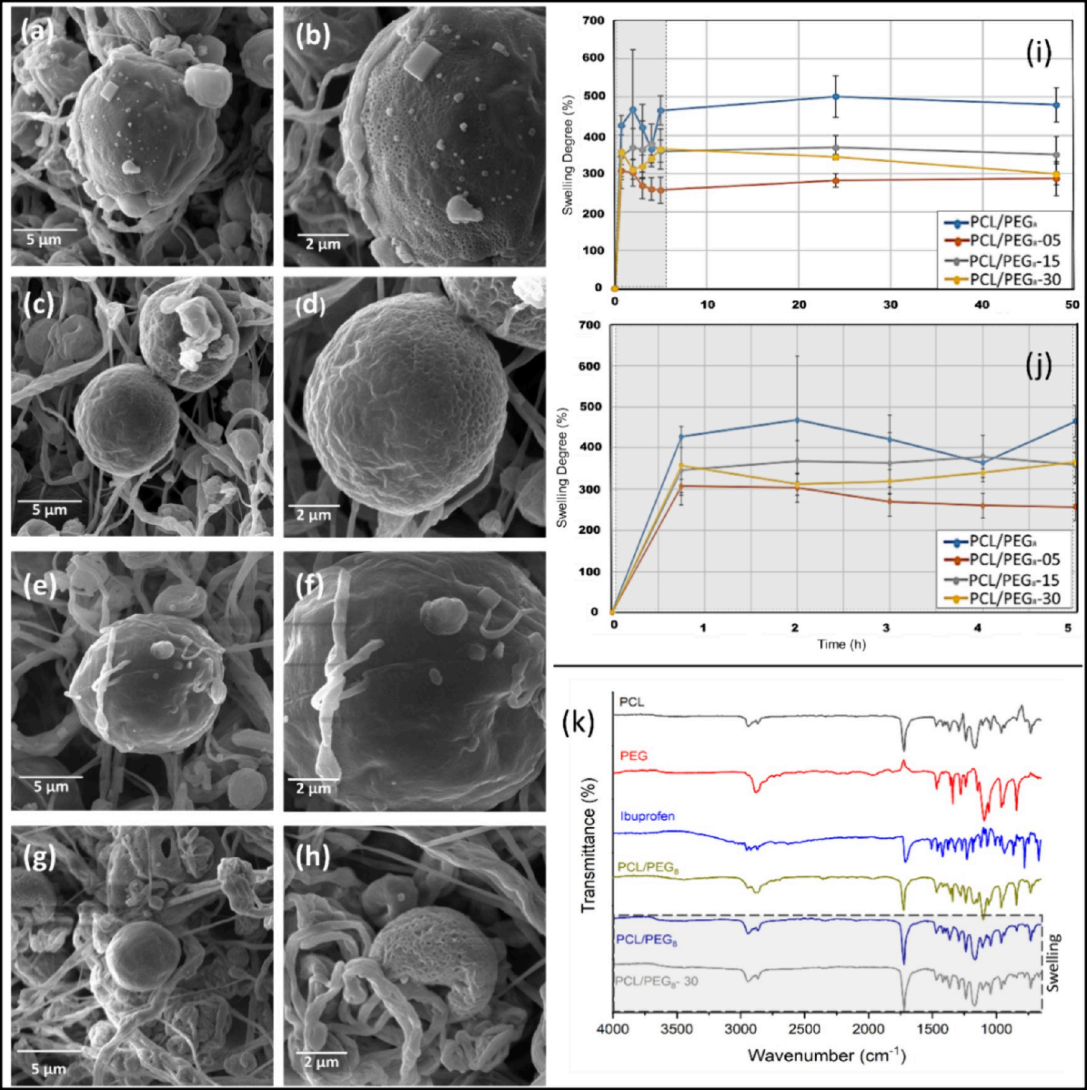
Degree of swelling on PCL/PEG_8_ mats with variation in the Ibuprofen concentration. SEM images of each sample spun by SBS assay variation ibuprofen after swelling degree with two increases (5k× and 10k×). Without ibuprofen - PCL/PEG_8_ (a, b); with 5 wt % of ibuprofen - PCL/PEG_8_-05 (c, d); with 15 wt % Ibuprofen - PCL/PEG_8_-15 (e, f); and 30 wt % Ibuprofen - PCL/PEG_8_-30 (g, h); (i) degree of swelling of PCL/PEG_8_-Ibuprofen mats after immersion in saline solution (NaCl 0.9 wt %) at 37 °C. (j) Highlighting the degree of swelling of PCL/PEG_8_-Ibuprofen films in the first 5 h of immersion in saline solution at 37 °C. (k) Evaluation by FTIR-ATR of the chemical composition of PCL/PEG_8_-Ibu mats after the swelling test.

The liquid adsorption capacity of a dressing is an essential characteristic when evaluating its effectiveness. It provides the necessary moisture conditions to maintain an optimal environment for cell proliferation and repair, while simultaneously removing purulent exudate from the wound. This process aids in the elimination of dead cells, blood, and toxins secreted by the injured tissue.^2^ In this regard, the liquid and/or exudate absorption capacity of wounds was evaluated using the PCL/PEG_8_ mat swelling test, as shown in Figure 7, using triplicate samples (*n* = 3) for each composition examined in saline solution (0.9 wt % NaCl). After the swelling test, the samples were morphologically characterized by SEM (Figure 7a–h).

In the literature, the poor liquid adsorption capacity of fibrous matrices made from pure PCL is documented. However, other studies have demonstrated a significant increase in regenerative processes in skin wounds when these matrices exhibit greater hydrophilicity. Therefore, this study investigated the incorporation of PEG as a humectant agent into the mats.^64−67^

Figure 7i illustrates the liquid adsorption processes of the PCL/PEG_8_ blend over a 48-h period. The swelling behavior of the PCL/PEG_8_ spun mats, both with and without ibuprofen, exhibits higher liquid adsorption capacity during the first 30 min of the analysis. This is evident in Figure 7j, where the PCL/PEG_8_ sample without ibuprofen displayed the highest degree of swelling (value). This could be attributed to the greater release of PEG to interact with the medium, given its higher affinity for the surrounding environment. On the other hand, samples containing ibuprofen exhibited lower liquid adsorption capacity.

Figure 7i shows the relationship between the degree of swelling for the mats over a 48-h period, with emphasis on the first 5 h of the experiment (Figure 7j). The liquid adsorption behavior of the mats revealed a high liquid absorption capacity for PCL/PEG_8_-ibuprofen beaded-fibers, with little variation in swelling throughout the 48-h test.

The peak of liquid absorption (physiological saline) was observed within the first 45 min of immersion for all samples, followed by an equilibrium profile. The data suggest that the spun mats containing ibuprofen exhibited a lower degree of swelling compared to neat PCL/PEG_8_ mats, possibly due to mass loss associated with PEG dissolution and drug release, since liquid absorption and drug release occur simultaneously.

All samples showed a decrease in the degree of swelling before the first 5 h of the test. To investigate the cause of this reduction, samples collected within this period were frozen, lyophilized for 24 h, and subsequently analyzed morphologically by SEM. The images revealed an increase in structure size compared to the preswelling test structures.^19,61^

On the other hand, a notable observation was the formation of pores, particularly in the microbeads, as seen in the SEM images in Figure 7. Other samples used in this analysis were chemically characterized by FTIR-ATR (Figure 7k). Here, the disappearance of the band at 2970 cm^–1^, associated with PEG, along with an increase in the intensity of the band at 1700 cm^–1^, suggests a chemical interaction between PEG and the surrounding medium (saline solution), leading to the dissolution of PEG within the nanostructured matrix.

The saline solution probably modifies the intermolecular interactions of PEG, disrupting its hydrogen bonding and ionic interactions, which may cause changes in the PEG crystalline or amorphous structure. This interaction promotes PEG dissolution or transformation within the matrix, potentially affecting its stability and behavior within the samples.

In short, this behavior suggests that encapsulated ibuprofen is predominantly localized within the microbeads of the material, which aligns with the findings in the literature on heterogeneous structures. Studies have shown that these microbeads act as reservoirs for materials and/or drugs, enabling a more efficient drug release compared to purely fibrous structures.

#### Ibuprofen Delivery Assay

**Figure 8:**
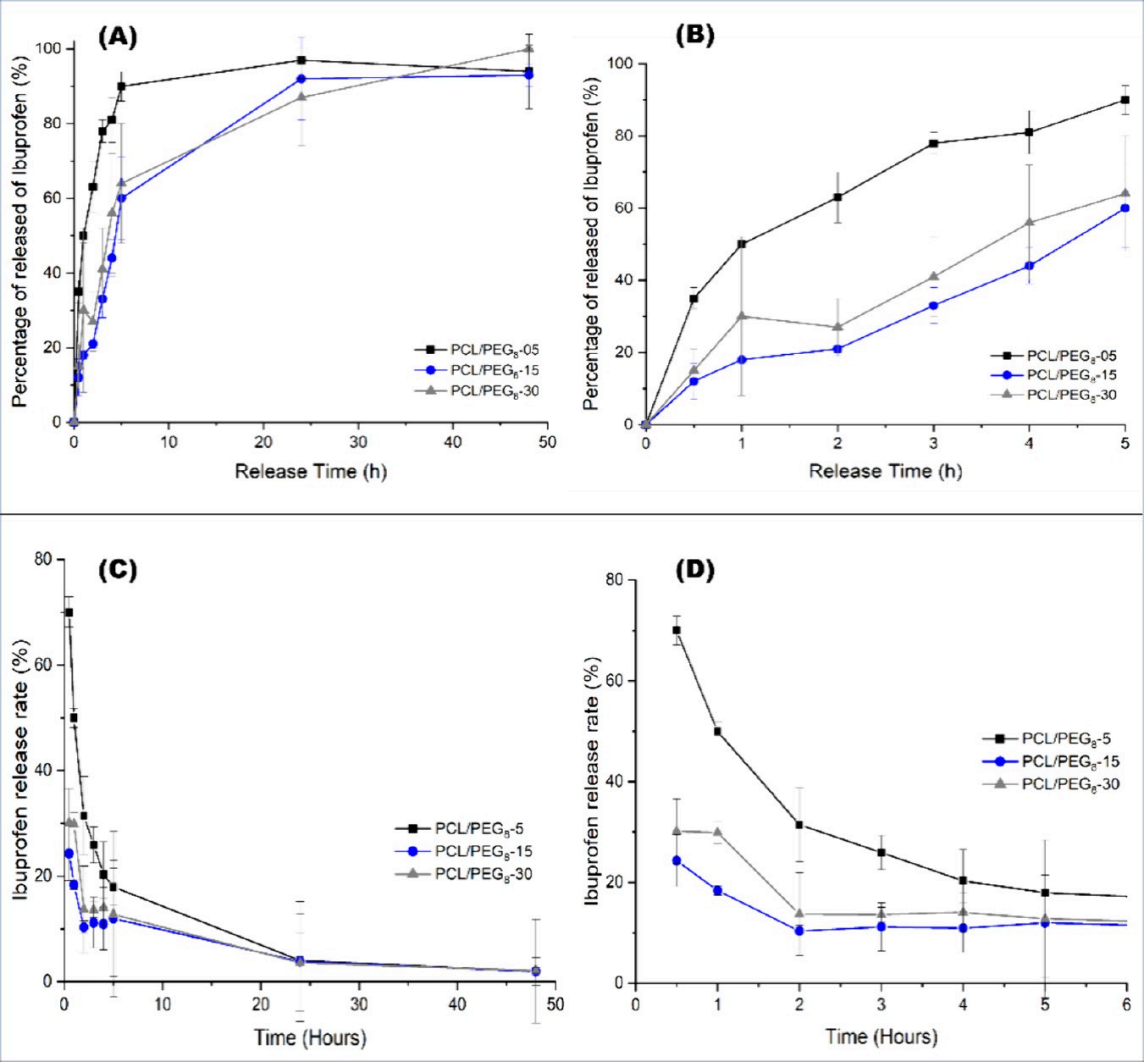
Delivery profile of ibuprofen from mats PCL/PEG_8_ beaded-fiber structures in saline solution (0.9% NaCl) at 37 °C (A), with highlight on the first 5 h of assay (B). Rate of Ibuprofen release from PCL/PEG_8_ beaded-fiber structures in saline solution at 37 °C by 48 h of assay (C), and (D) Release behavior in the 5 first hour of assay.

Figure 8 depicts the ibuprofen release process. Absorbance values were measured using UV–vis spectroscopy, following the data range established by the drug supplier, in which absorption maxima were observed at 265 and 273 nm. These values are also found in the literature for evaluating ibuprofen release.^51^

Figure 8 details the ibuprofen release rate from each heterogeneously structured PCL/PEG_8_ spun-bond nonwoven mat obtained via SBS. However, the ibuprofen concentration released into the medium exceeded the estimated theoretical value based on TGA analysis (Table 3).

Therefore, it can be assumed that the drug exhibited a heterogeneous distribution throughout the nonwoven structure. These values are shown in Figure 8, presented in relation to the total amount of ibuprofen released by the nonwovens over the 48-h evaluation period.

Additionally, the PCL/PEG_8_ nonwovens without ibuprofen were also evaluated to assess any potential interference from the solvent or polymer degradation in the medium. The values obtained from these control samples were subtracted from those of the ibuprofen-loaded nonwovens.

Analyzing the release profile for all the studied samples varying the encapsulated ibuprofen content (Figure 8), we observe an initial burst release, a common phenomenon in drug release profiles, followed by a more controlled release, maintaining a nearly constant rate after 24 h.

The PCL/PEG_8_-05 curve shows that a large portion of the release occurred within the first 5 h, followed by a stable ibuprofen concentration in the subsequent time points. The rapid initial release accounted for the release of approximately 90% of the drug contained in the mat.

Figure 8C,D illustrated the ibuprofen release rate from PCL/PEG_8_ beaded-fiber structures over time. The release rate was used to better describe the percentage of ibuprofen released as a function of time. A burst release was observed in the initial drug release profile, followed by a gradual decrease in the release rate. Nearly all of the ibuprofen content was released within the first 5 h. After this period, approximately 20% of the remaining drug was slowly released over time.

The PCL/PEG_8_-15 and PCL/PEG_8_-30 curves exhibited a similar ibuprofen release behavior throughout the entire release assay (Figure 8A). Initially, both samples displayed a lower initial release rate during the first 5 h, compared to the PCL/PEG_8_-05 sample, taking longer to reach a stability plateau. Drug release stabilized between 2 and 5 h, with approximately 60% of the ibuprofen being released.

Although the PCL/PEG_8_-30 sample (30 wt % ibuprofen) exhibited a more linear release profile after 5 h, its overall release rate remained very similar to that of the PCL/PEG_8_-15 sample throughout the entire assay. This suggests that, despite the presence of more microbeads in the PCL/PEG_8_-30 sample, which may have contributed to a more sustained release, the final release rates for the 15 and 30% ibuprofen-loaded mats were comparable.

These findings demonstrate that morphology influences drug release kinetics, although the total release rates for PCL/PEG_8_-15 and PCL/PEG_8_-30 remained similar, in agreement with previously reported findings in the literature.^10,68^

Studies on nonsteroidal drug delivery systems have primarily focused on polymeric matrices composed solely of nanofibers. These studies have consistently reported a burst release, with approximately 78% of the drug being delivered within the first 2 h of testing.

In their study, Potrc et al.^69^ electrospun PCL nanofibers containing 5 to 30 wt % ibuprofen (based on the dry weight of PCL), obtaining nanofibers without the presence of beads, where complete release (∼96%) was observed within the first 4 h of testing. This finding reinforces the hypothesis that beads act as ibuprofen reservoirs and release controllers. However, the observed values remain within the error range. From the release test, it was possible to demonstrate the potential of these mats for the controlled release of ibuprofen.

Fernández-Carballido et al.^70^ calculated the theoretical therapeutic concentration of 8 μg/mL based on the pharmacokinetic parameters of ibuprofen, i.e., the study of the drug’s effect on the body. We observed that the theoretical therapeutic concentration of ibuprofen was achieved by the PCL/PEG_8_-Ibu fibers in the first 30 min, so it has the potential to act in the early stages of inflammatory processes.

Sigg^71^ investigated the encapsulation of ibuprofen in PLA nanofibers, which had diameters below 120 nm and exhibited a 76% drug release within the first hour of testing. Similarly, Dziemidowicz et al.^72^ electrospun nanofibrous PCL mats with diameters ranging from 200 to 300 nm, loaded with ibuprofen. The authors found that these fibers released up to 78% of the drug within the first hour of testing.

Gao et al.^73^ produced electrospun nanofiber matrices composed of PVP and mesoporous silica as a drug delivery system. The resulting fibers had diameters ranging from 200 nm to 2 μm, with the majority of the drug being released within the first 2 h. The authors attributed the more sustained release observed in the later stages to the presence of silica particles, which contributed to a slower release profile compared to pure nanofibers.

Following these findings in the literature, it is evident that drug release rates depend not only on the polymer type but also on fiber diameter. These studies demonstrate that nanofibers with diameters below 300 nm exhibit burst release within the first hour, whereas fiber-based delivery systems with diameters exceeding 300 nm achieve extended-release times beyond 2 h.

In contrast, our beaded nanofiber system significantly extended the ibuprofen release time to approximately 5 h, representing a more than 150% increase compared to the fiber-only systems reported in the literature. This prolonged release can be attributed to the presence of microbeads within the nanofibrous matrix, which could act as additional reservoirs, enabling a more sustained drug release profile.^74^

The primary objective of this study was the encapsulation of ibuprofen in PCL/PEG_8_ matrices, with a focus on evaluating the processing, encapsulation efficiency, and release behavior. However, the handling properties of these materials are a critical factor for their potential application in skin dressings. Based on the obtained results, we decided to proceed with PCL/PEG formulations without ibuprofen at concentrations of 5 and 15%. These formulations exhibited improved structural stability and reproducibility, as well as enhanced ease of characterization and handling. As a result, they are deemed more suitable for application in skin dressings.

### In Vitro Assay

#### Cell Viability

As a first approach to investigate the potential cytotoxic effects of the ibuprofen-loaded PCL/PEG_8_ mats, we evaluated the impact of the direct placement of these membranes over a 2D cell culture. Two spun mat samples were tested, using PCL/PEG_8_ as reference, while the sample PCL/PEG_8_-15 was selected due to its better swelling performance and subsequent ibuprofen release, with values lower than the therapeutic dose, relative to the formulation studied in this work.

**Figure 9:**
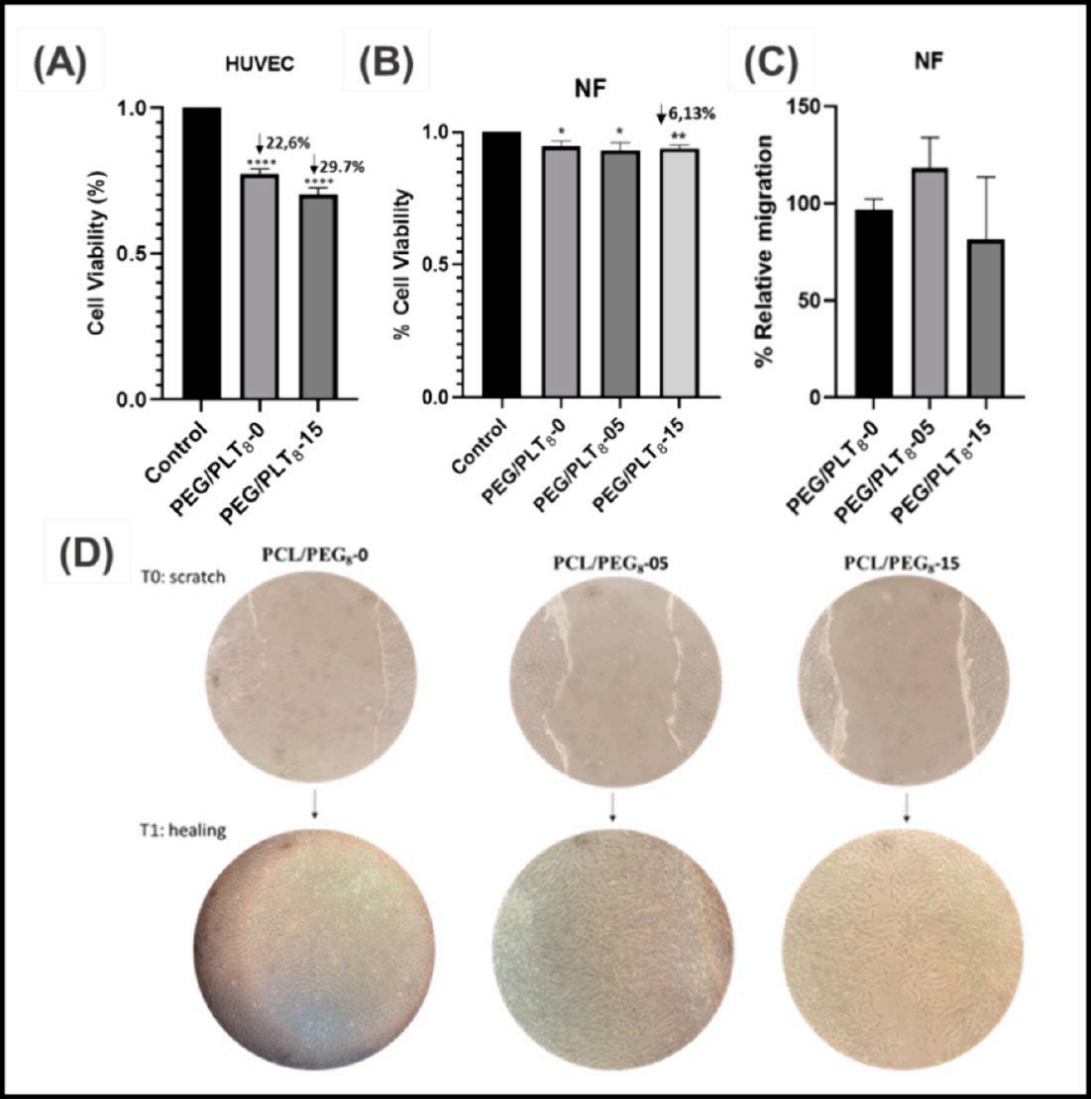
Effect in healthy cell viability and migration capacity of NF PCL/PEG_8_ mats loaded with different amounts of ibuprofen. (A) MTT test of HUVEC cells on PCL/PEG_8_-IBU deposited on the well, (B) viability of Normal fibroblasts. *P < 0.05, **P < 0.01, ****P < 0.0001 from Control (HUVEC cells without PEG/PCL8 mat incubation, *n* = 3); (C) representative image of the experiment at T0 (wound) or after 24 h at the T1 (healing). (D) Ratio between the surface of the gap at the start and the same surface at the end of the experiment and are expressed as the percentage of relative migration of NF in contact with each PCL/PEG_8_ mats. (*n* = 3). ns = not statistically significant.

For this, we first selected the endothelial-like cell line HUVEC as a model of nontransformed cells. As shown in Figure 9A, direct contact between the ibuprofen-loaded mats (PCL/PEG_8_-0 and PCL/PEG_8_-15) and HUVEC cells resulted in only a modest reduction in cell viability (17.3 and 24.5%, respectively, compared to the negative Control), indicating the low toxicity associated with these mats. This result was compared with the viability of HUVEC cells treated with ibuprofen.^75^

The results of this experiment might have been affected by potential disturbances in the cell culture conditions, induced by the direct interaction between the mats and the cells. However, it is important to highlight that the international standard ISO 10993-5 (Biological evaluation of medical devices – Part 5: Tests for in vitro cytotoxicity) classifies a sample cytotoxic if it reduces cell viability by more than 30%. Thus, according to this criterion, PCL/PEG_8_-IBU mats are not cytotoxic.

Despite this, we designed a complementary experimental approach to evaluate the effect of compounds released by the PCL/PEG_8_ mats, avoiding direct contact with the cultured cells. To achieve this, we used Boyden chambers, allowing a physical separation between the PCL/PEG_8_-IBU mats (placed in the upper compartment) and the cell line of interest (normal nontransformed fibroblasts, NF) cultured in the lower compartment.

Using this approach, we found that NF viability was minimally affected by the ibuprofen released from the polymeric mats, with a maximal reduction of cell viability of 6.13% of the highest ibuprofen concentration used (Figure 9B).

#### Wound Healing

To further confirm these observations, we analyzed the impact of the ibuprofen-loaded PCL/PEG_8_ mats on the migratory capacity of NF using the wound-healing assay. This assay determines the ability of cells to fill the gap (wound) created in a cell monolayer (Figure 9C,D). In this experiment, we placed the PCL/PEG_8_–IBU mats over the wound, allowing us to investigate whether the presence of ibuprofen in each polymer mat could stimulate NF migration and consequently accelerate healing.

As shown in Figure 9C, the impact on healthy cell viability and migration capacity did not reveal significant differences in the ability of NF cells to bridge the gap when exposed to PCL/PEG_8_ mats. It was observed that the quantity of the drug had no significant influence on cell migration over the 24-h assay period, as complete migration was achieved for all three evaluated samples, both with and without ibuprofen. These findings align with studies reported in the literature on electrospun systems loaded with anti-inflammatory drugs, which also achieved favorable migration outcomes within 24 h for low drug concentrations (∼4 wt %).^76^ Similar results were observed for mats with beaded-fiber morphology loaded with propolis, promoting efficient proliferation of FGH cells.^10^

#### Protein Expression Analysis

**Figure 10:**
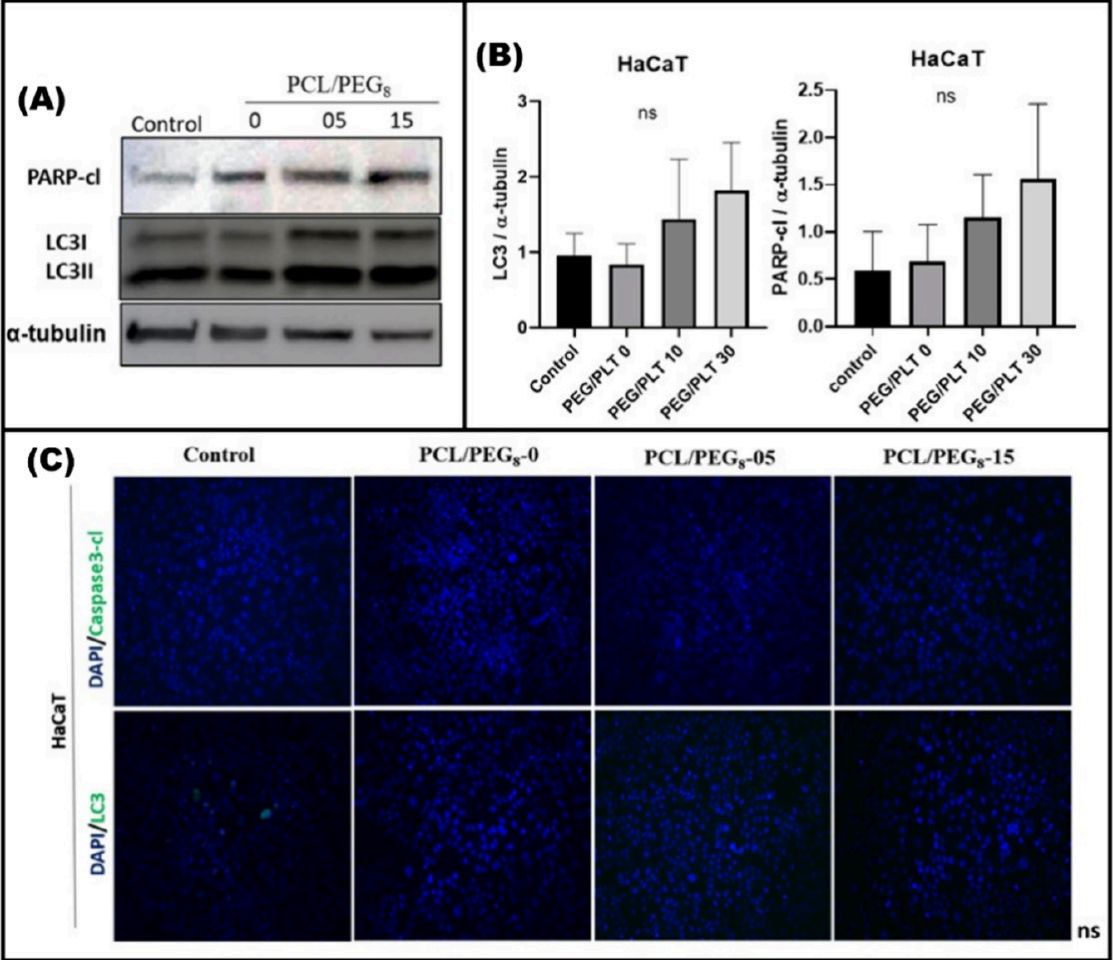
PCL/PEG_8_ mat incubation in HaCaT cells did not affect cell death protein expression levels. (A) Effect of the different PCL/PEG_8_ mats incubation with different amounts of ibuprofen during 48 h on PARP-cleaved and LC3I/II levels. A representative Western blot of a *n* = 3 is shown. (B) Data correspond to the densitometric analysis of the levels of each protein relative to negative control cells, they are expressed as the mean fold change ± SEM (*n* = 3). (C) Effect of incubation with PCL/PEG8-0, 05, and 15 for 48 h on LC3 and Caspase3-cleaved immunofluorescence in HaCaT cells. Representative images are shown (*n* = 1). ns = not statistically significant.

To complete the analysis of the effect of PCL/PEG_8_ mats on the viability of nontransformed cells, we evaluated whether incubation with these beaded-fiber mats induced apoptosis or autophagy, two processes associated with cytotoxicity, in the HaCat cell line (immortalized keratinocytes). To do this, we analyzed the expression of cleaved-PARP, as PARP is a substrate of the executioner caspases Caspase 3/7, and its cleavage is considered a hallmark of apoptosis. Additionally, we examined the presence of LC3-II, the lapidated, autophagosome-associated form of the autophagic protein LC3, which is regarded as a hallmark of autophagy. As shown in Figure 10, incubation with PCL/PEG_8_ mats did not significantly affect PARP cleavage or LC3 lipidation.

It can therefore be postulated that the ibuprofen-loaded PCL/PEG_8_ mats did not significantly trigger HaCaT cell death. In summary, these in vitro assays were conducted to determine whether ibuprofen-loaded PCL/PEG_8_ mats could cause a change in the viability of healthy cells (as similar as possible to skin cells) and, if so, whether this potential cytotoxicity was related to the activation of molecular cell death mechanisms.

Cell viability was found to be altered as the percentage of ibuprofen in the PCL/PEG_8_ mats increased. However, despite this decrease in viability, while significantly different from the control, it was not considerably high and could be acceptable in this context.

In addition, to investigate whether cell death mechanisms were being activated, several experiments were performed, allowing us to verify that healthy cells did not activate these mechanisms even at a high ibuprofen concentration in the PCL/PEG_8_ beaded-fiber mats, corroborating findings reported in the literature.^77^

## Conclusions

In this study, heterogeneous structured mats were prepared using solution blow spinning (SBS) with analgesic action for the treatment of skin injuries, providing patient comfort through the release of ibuprofen. This study demonstrated that the optimal PCL/PEG mass ratio was 1:1, due to the processability and morphology of the blend, which generated homogeneous nanofibers (∼510 nm) with more spherical and uniform micrometric beads of an approximate average diameter of 3.9 ± 2.5 μm. Additionally, it was observed that the incorporation of ibuprofen resulted in a higher number of defects in the beaded-fiber structure, with an increase in the drug concentration. For all the studied formulations, an initial ibuprofen release profile of 70% within the first 5 h was observed when exposed to saline solution (0.9 wt % NaCl), demonstrating longer drug delivery times compared to pure fiber matrices (∼2 h).

On the other hand, SEM analysis of the mats after swelling/release tests confirmed the encapsulation of ibuprofen, mainly within the microbeads of the beaded-fiber structure. These analyses demonstrated the delivery of PEG-ibuprofen, leaving the microbeads porous and with an increased capacity of exudate adsorption. The results obtained in this work suggest that ibuprofen was heterogeneously encapsulated within the matrix structure.

However, the ibuprofen-loaded mats exhibited an increased liquid absorption capacity, with a degree of fiber swelling in saline solution at 37 °C ranging from 250 to 380%. A gradual release of ibuprofen was observed within the first 5 h of immersion for all mats. After evaluation for the potential use of PCL/PEG-Ibu beaded-fibers for skin dressings, it was concluded that the PCL/PEG_8_-15 mat is the recommended base for future studies using this system. In addition to exhibiting the highest degree of swelling among the samples, it showed a more gradual ibuprofen release profile and less dispersion of the fiber diameters.

Finally, in vitro analyses demonstrated that the PCL/PEG_8_-Ibu mats did not activate programmed cell death pathways such as apoptosis or autophagy. Instead, they altered the cellular microenvironment and influenced some cellular responses that could indirectly affect viability, but not through a programmed cell death mechanism. Thus, despite causing a reduction in cell viability, PCL/PEG_8_-Ibu mats exhibited viability values within an acceptable range for their application in transdermal dressings for skin wounds, demonstrating potential for accelerating wound healing and providing pain relief for the patient for 5 h. Moreover, PCL/PEG_8_ beaded-fibered mats produced by the SBS technique proved to be suitable vehicles for encapsulating NSAIDs and hydrophobic drugs.

## Supporting Information Available

Comparative TGA analyses of solution blow spun samples containing 5 and 15 wt % ibuprofen (PCL/PEG_8_-05 and PCL/PEG_8_-15, respectively) (PDF)

## Notes

The authors acknowledge Coordenação de Aperfeiçoamento de Pessoal de Nivel Superior–CAPES, the National Council for Scientific and Technological Development–CNPq (Grant 308789/2020-6), and Fundação Carlos Chagas Filho de Amparo à Pesquisa do Estado de Rio de Janeiro–FAPERJ (Grant: NanoSaúde–E-26/210.139/2019) for financial support.
