# Supplementary material for: Solution Blow Spun Mats with Beaded-Fiber Morphologies as a Drug Delivery System with Potential Use for Skin Wound Dressing
Source: ACS Appl Mater Interfaces. 2025 Apr 10;17(16):23466–83. doi: 10.1021/acsami.4c16675 (PMC12022951; doi:10.1021/acsami.4c16675)
Supplement: Supplementary file 1 — am4c16675_si_001.pdf [file am4c16675_si_001.pdf]

Supporting Information

Solution Blow Spun Mats with Beaded-Fiber Morphologies as a Drug Delivery System with Potential Use for Skin Wound Dressing

Javier Mauricio Anaya-Mancipe,[1] *Aline Luiza Machado Carlos,*[1] *João Victor Dias de Assumpção Bastos,*[1] *Elena Maria Tovar Ambel,*[2,3] *Guillermo Velasco-Díez,*[2,3] *Rosana Lopes Fialho,*[4] *Rossana Mara da Silva Moreira Thiré.*[1]*

[1] Program of Metallurgical and Materials Engineering – PEMM/COPPE, Universidade Federal do Rio de Janeiro (UFRJ), Rio de Janeiro, 21941-598 RJ, Brazil.

[2] Department of Biochemistry and Molecular Biology, School of Biology, Universidad Complutense de Madrid – UCM, 28040, Madrid, Spain.

[3] Instituto de Investigaciones Sanitarias San Carlos (IdISSC), 28040, Madrid, Spain.

[4] Postgraduation Program in Industrial Engineering, Polytechnic School, Universidade Federal da Bahia (UFBA), 40210-630, Salvador BA, Brazil.

*Corresponding Author: rossana@metalmat.ufrj.br

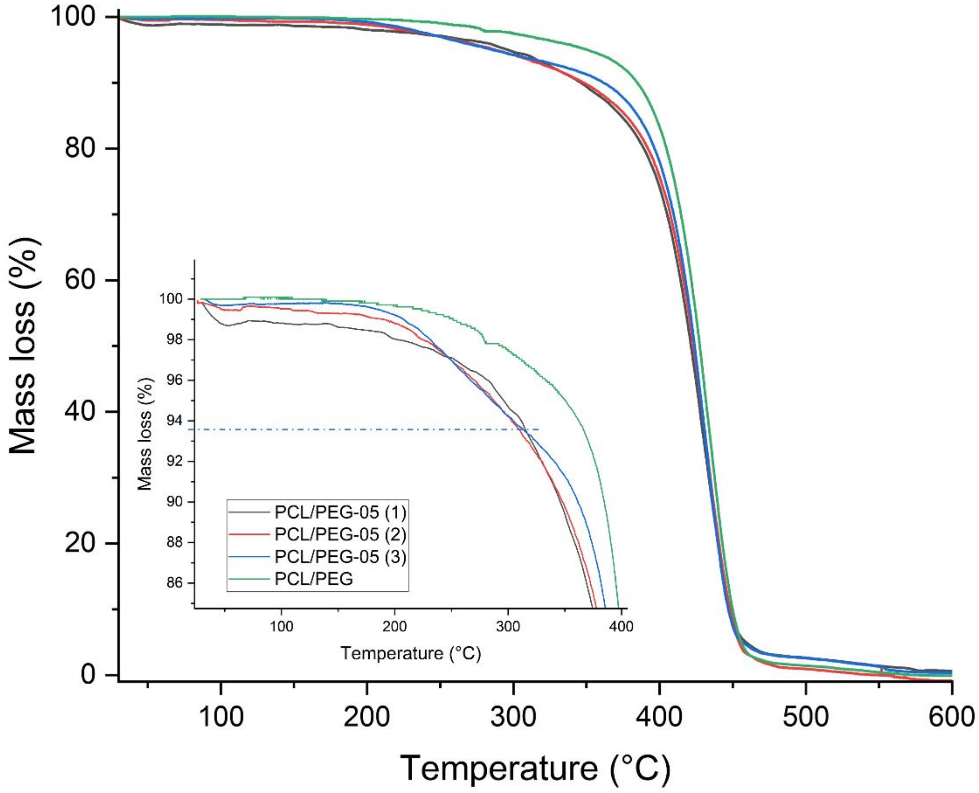

Figure S1. Comparative TGA thermogram for Beaded-fibers mats of PCL/PEG_8-05.

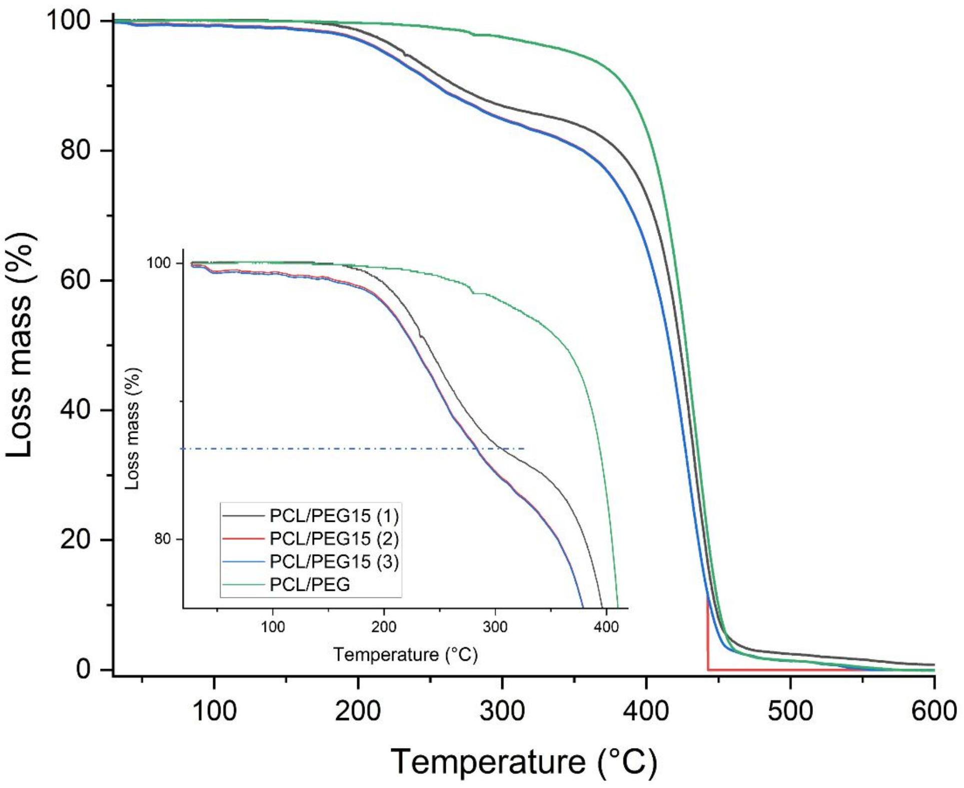

Figure S2. Comparative TGA thermogram for Beaded-fibers mats of PCL/PEG_8-15.
